# Hydroxygenkwanin Suppresses Non-Small Cell Lung Cancer Progression by Enhancing EGFR Degradation

**DOI:** 10.3390/molecules25040941

**Published:** 2020-02-19

**Authors:** Yann-Lii Leu, Tong-Hong Wang, Chih-Ching Wu, Kuo-Yen Huang, Yu-Wen Jiang, Yi-Chiung Hsu, Chi-Yuan Chen

**Affiliations:** 1 Graduate Institute of Natural Products, Chang Gung University, Taoyuan 333, Taiwan; ylleu@mail.cgu.edu.tw; 2 Center for Traditional Chinese Medicine, Chang Gung Memorial Hospital at Linkou, Taoyuan 333, Taiwan; 3 Graduate Institute of Health Industry Technology and Research Center for Food and Cosmetic Safety, Research Center for Chinese Herbal Medicine, College of Human Ecology, Chang Gung University of Science and Technology, Taoyuan 333, Taiwan; cellww@gmail.com (T.-H.W.); sjiang0715@gmail.com (Y.-W.J.); 4 Tissue Bank, Chang Gung Memorial Hospital at Linkou, Taoyuan 333, Taiwan; 5 Department of Medical Biotechnology and Laboratory Science, College of Medicine, Chang Gung University, Taoyuan 333, Taiwan; luckywu@mail.cgu.edu.tw; 6 Department of Otolaryngology-Head&Neck Surgery, Chang Gung Memorial Hospital at Linkou, Taoyuan 333, Taiwan; 7 Institute of Biomedical Sciences, Academia Sinica, Taipei 115, Taiwan; kyhuang0222@gmail.com; 8 Department of Biomedical Sciences and Engineering, National Central University, Taoyuan 320, Taiwan

**Keywords:** *Daphne genkwa*, hydroxygenkwanin, NSCLC, EGFR, apoptosis

## Abstract

Epidermal growth factor receptor (EGFR) is frequently overexpressed and mutated in non-small cell lung cancer (NSCLC), which is the major type of lung cancer. The EGFR tyrosine kinase inhibitors (TKIs) are the approved treatment for patients harboring activating mutations in the EGFR kinase. However, most of the patients treated with EGFR-TKIs developed resistance. Therefore, the development of compounds exhibiting unique antitumor activities might help to improve the management of NSCLC patients. The total flavonoids from *Daphne genkwa* Sieb. et Zucc. have been shown to contain antitumor activity. Here, we have isolated a novel flavonoid hydroxygenkwanin (HGK) that displays selective cytotoxic effects on all of the NSCLC cells tested. In this study, we employed NSCLC cells harboring EGFR mutations and xenograft mouse model to examine the antitumor activity of HGK on TKI-resistant NSCLC cells. The results showed that HGK suppressed cancer cell viability both in vitro and in vivo. Whole-transcriptome analysis suggests that EGFR is a potential upstream regulator that is involved in the gene expression changes affected by HGK. In support of this analysis, we presented evidence that HGK reduced the level of EGFR and inhibited several EGFR-downstream signalings. These results suggest that the antitumor activity of HGK against TKI-resistant NSCLC cells acts by enhancing the degradation of EGFR.

## 1. Introduction

The epidermal growth factor receptor (EGFR) pathway is one of the most dysregulated molecular pathways in human cancers. The activating mutations of EGFR occur in approximately ~10% of non-small cell lung cancer (NSCLC) cases in in North America and Western Europe patients and approximately 30–50% in East Asian patients [1]. These tumors are oncogene-addicted to EGFR-mediated survival pathway and they are highly sensitive to apoptosis induction by tyrosine kinase inhibitors (TKIs) [2]. The TKIs are approved treatments for patients harboring activating mutations in the EGFR kinase. Several generations of TKIs have been developed and these drugs are superior to conventional chemotherapy in prolonging progression-free survival (PFS) of NSCLC patients harboring common EGFR mutations, including exon 19 deletions (Del19) and L858R [3]. However, most of the patients treated with TKIs developed resistance within 9–14 months [4,5]. Therefore, the development of compounds exhibiting unique pharmacologic properties and antitumor activities for NSCLC patients at high risk of recurrence is urgently needed to help in the management of NSCLC patients.

*Daphne genkwa* Sieb.et Zucc. has been used in traditional Chinese medicine for thousands of years. The flower buds of this plant (“Genkwa Flos”) are mainly used for the treatment of cancer, asthma, and edema [6,7,8,9]. It contains several types of compounds, including flavonoids, biscoumarin, lignans, volatile oils, diterpene esters, chlorogenic acids, and phenolic glycosides. The flavonoids and diterpene esters are thought to be the major efficacy components [10,11]. Yuanhuadine, a Daphnane diterpene from Genkwa Flos, has been reported to inhibit the growth of human lung cancer cells, which was accompanied with cell cycle arrest, up-regulation of p21, and down-regulation of c-Myc, CDK2, CDK4, and cyclins [12]. Yuanhuadine also inhibits ligand-induced EGFR and c-Met signaling [12]. Yuanhuacine, a Daphnane diterpenoid from Genkwa Flos, has been shown to modulate the AMPK/mTORC2 signaling pathway and actin cytoskeleton organization in NSCLC cells [13]. The total flavonoids from Genkwa Flos have been shown to inhibit the growth of Lewis lung carcinoma in C57BL6 mice and colorectal cancer cells [14,15]. However, the active components in the flavonoids of Genkwa Flos have not been characterized. In this study, we have identified hydroxygenkwanin (HGK) as one of the active flavonoids that display anti-tumor activity against TKI-resistant NSCLC cells in vitro and in vivo.

## 2. Results

### 2.1. Isolation and Identification of Flavonoids from Genkwa Flos

**Figure 1:**
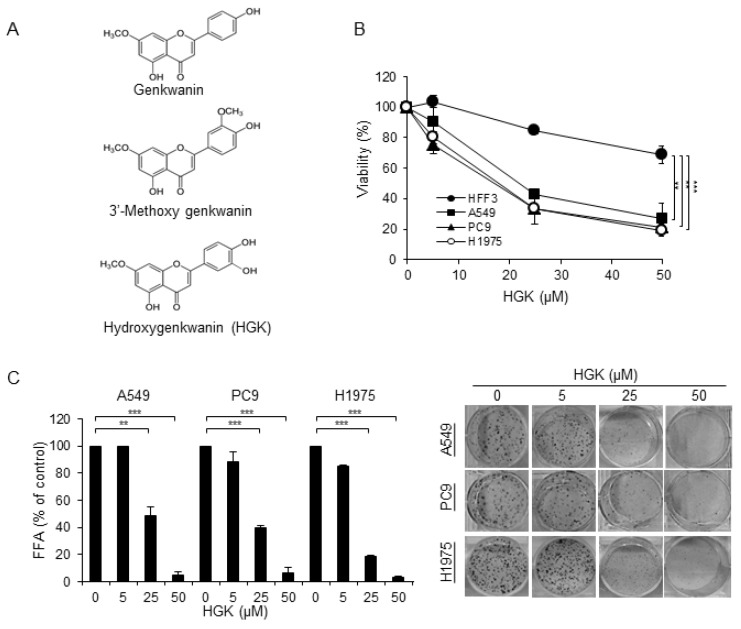
Effects of hydroxygenkwanin (HGK) on the viability of non-small cell lung cancer (NSCLC) cells. (**A**) Structures of genkwanin, 3′-methoxy genkwanin, and HGK. (**B**) Cells were treated with various concentrations of HGK for 24 h and the viability of treated cells was evaluated by staining with trypan blue. (**C**) Cells were treated with various concentrations of HGK for 24 h and then cultured in the absence of HGK for an additional five days. The number of foci was scored, and the data are presented as relative focus-forming ability (FFA). Data are expressed as mean ± SD of three independent experiments. ** *p* < 0.01; and *** *p* < 0.001, as analyzed with the unpaired *t*-test.

Genkwa Flos were extracted with methanol and then concentrated to give brown syrup. The syrup was partitioned first in CHCl_3_/water (1:1) and then in *n*-butanol/water (1:1) [16]. The cytotoxic effects of the fractionated extracts on lung cancer cells A549 were investigated by treating the cells with extracted compounds at 30 μg/mL for 48 h and the cell viability was determined by MTT assay. The chloroform fraction had the highest cytotoxic effects in A549 cells (viability 38.50%) as compared with the water (viability 99.84%) or *n*-butanol fractions (viability 75.38%), which suggested that the anti-NSCLC agents are mainly contained in the chloroform fraction. To identify and isolate the active compounds that display anti-tumor activity, the chloroform fraction was subjected to silica gel column chromatography (see Materials and Methods) in order to obtain three known flavonoid compounds (Figure 1A), including genkwanin, 3-methoxy genkwanin, and hydroxygenkwanin (HGK). The identity of these three compounds has been confirmed by comparing their UV, IR, NMR, and MS data with those of authentic compounds and/or with those that were reported in the literature [10,17,18].

The three flavonoid compounds that were isolated from the chloroform fraction of Genkwa Flos extract were first tested for their selective cytotoxicity against lung cancer cells. The lung cancer cells A549 and normal fibroblasts HFF3 were treated with each compound for 24 h and then assayed for viability by MTT assay. The HGK exhibited the strongest selective cytotoxicity against lung cancer cells, as shown in Table 1. The half maximal inhibitory concentration (IC50) of HGK for A549 cells was about 22 μM (Figure 1B), while the IC50s of genkwanin and 3-methoxy genkwanin against A549 cells were >100 μM. A search of the literature has revealed that HGK that was isolated from the leaves of *Blumea balsamifera* DC displayed strong cytotoxicity against human lung cancer cells (NCI-H187) and a moderate toxicity against oral cavity cancer cells lines (KB) [19]. HGK induced DNA damage, cell cycle arrest, and cell apoptosis in glioma [20]. HGK inhibited cell migration, invasion, and proliferation in oral squamous cell carcinoma and hepatocellular carcinoma [21,22]. These data suggest that HGK is one of the active antitumor flavonoids in the Genkwa Flos extract. 

### 2.2. The Cytotoxic Effect of HGK

The cytotoxic effects of HGK on PC9 (EGFR Del19, TKI-sensitive), and H1975 (EGFRL858R/T790M, TKI-resistant) were compared to that of a NSCLC cell line A549 and normal fibroblasts HFF3 cells to evaluate whether HGK displays antitumor effects against NSCLC cells harboring constitutive activating EGFR mutations. As shown in Figure 1B, HGK displayed greater cytotoxic effects on all three lung cancer cells than the normal fibroblasts HFF3. The half maximal inhibitory concentration (IC50) of HGK for A549, PC9, and H1975 cells were 22.0 ± 0.9 μM, 18.3 ± 3.1 μM, and 18.3 ± 0.3 μM, respectively. Since the A549 cells that were treated with HGK at 100 μM (Table 1) had a similar survival as that treated at 50 μM (Figure 1B), this suggests that the cytotoxic effects of this drug peaked at around 50 μM. Therefore, the highest dose of HGK used in all of the subsequent experiments was set at 50 μM. Next, we examined the effects of HGK on the foci formation of NSCLC cells. As shown in Figure 1C, HGK inhibited foci formation in a concentration-dependent manner in all three NSCLC cells. Together, these results indicate that the TKI-sensitive and TKI-resistant NSCLC cells are all sensitive to the treatment of HGK. 

### 2.3. Effects of HGK on the Cell Cycle Progression and Apoptosis

**Figure 2:**
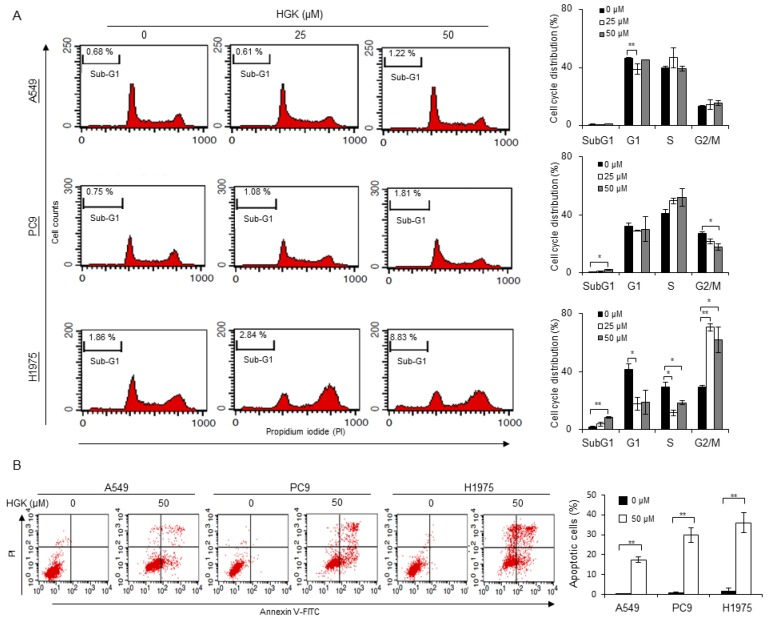
Effects of HGK on cell cycle and apoptosis in NSCLC cells. Cells were treated with HGK at the indicated concentrations for 24 h. (**A**) The distribution of cells in different phases of cell cycle was analyzed by flow cytometry of cells stained with propidium iodide. (**B**) Apoptosis was detected by flow cytometry of cells stained with Annexin V-FITC. The data shown are expressed as the mean ± SD of three independent experiments. Symbols: * *p* < 0.05 and ** *p* < 0.01, as analyzed by unpaired *t*-tests.

**Figure 3:**
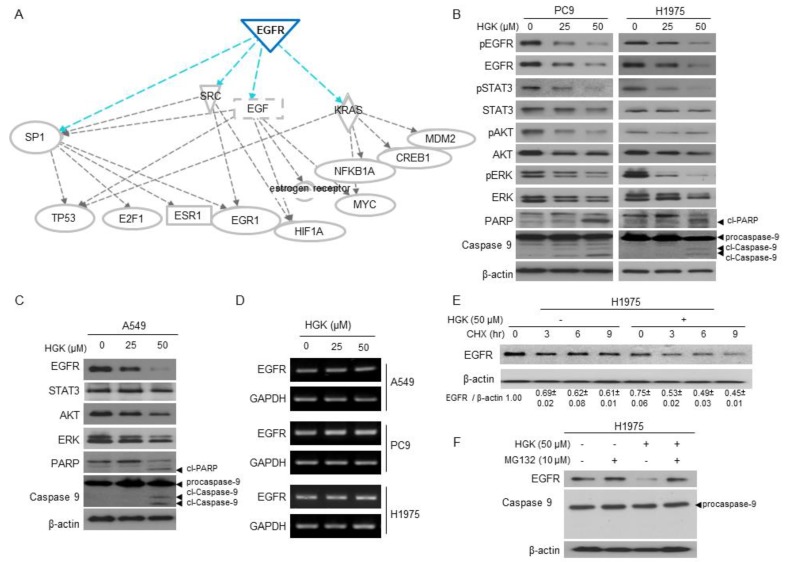
Effects of HGK on epidermal growth factor receptor (EGFR) signaling pathway in NSCLC cells. (**A**) The prediction of EGFR as the upstream transcriptional regulators by using the Ingenuity Pathway Analysis (IPA). (**B** and **C**) Cells were treated with indicated concentrations of HGK for 24 h. The expression levels of phosphor-EGFR (pEGFR), EGFR, phospho-STAT3 (pSTAT3), phosphor-AKT (pAKT), AKT, phosphor-ERK (pERK), ERK, as well as the level of cleaved poly (ADP-ribose) polymerase (cl-PARP) and cleaved caspase 9 (cl-caspase 9) were determined by Western blot. β-actin served as a loading control. (**D**) The expression level of EGFR mRNA in HGK-treated cells was determined by RT-PCR. GAPDH served as the loading control. (**E**) H1975 cells were incubated with cycloheximide (CHX) 100 mg/mL for the indicated times in absence or presence of HGK 50 μM. The relative expression levels of EGFR were quantified and shown at the bottom. (**F**) H1975 cells were incubated with HGK in the presence or absence of MG132 for 12 h. The expression levels of EGFR and caspase 9 were analyzed by Western blot. β-actin used as a loading control. The data shown in (**A**) to (**F**) are from one of three similar results.

The effects of HGK on cell cycle progression and apoptosis were examined to investigate the cytotoxic mechanism of HGK. For cell cycle analysis, the NSCLC cells were treated with HGK for 24 h and examined by flow cytometry using propidium iodide (PI) staining. As shown in Figure 2A, the percentage of treated-cells in the sub-G1 region was only significantly increased in the H1975 cells that were treated with HGK. An accumulation of cells in G2/M phase, and reduced distribution in the G0/G1 and S phase, were also only observed in HGK-treated H1975 cells, but not the HGK-treated A549 or PC9 cells. Therefore, HGK appears to affect cell cycle progression only in H1975 cells, despite HGK having similar cytotoxic effects on the three NSCLC cells (Figure 1B,C). To determine whether HGK induces apoptosis, the phosphatidylserine exposure in the cell surface and the cleavage of PARP and caspase 9 were examined. As shown in the upper and lower right quadrants of Figure 2B, cells that were positively stained with Annexin V-FITC were readily detected in all of the NSCLC cells treated with HGK at 50 μM for 24 h. Similarly, the cleavage of PARP and caspase 9 were readily detected in the HGK-treated NSCLC cells (Figure 3B,C). These data suggest that the cytotoxic effect of HGK is mediated through its induction of apoptosis.

### 2.4. The Molecular Mechanism of Antitumor Activity by HGK in H1975 Cells

We employed whole-transcriptome sequencing to analyze the effects of HGK on gene expression to further explore the mechanism of the action affected by HGK on H1975 cells. A total of 2345 putative genes showed a statistically significant two-fold difference in the expression level of H1975 cells that were treated with HGK at 50 μM when compared to the untreated cells. Next, we used the Ingenuity Pathway Analysis (IPA) for Canonical pathway analysis to conduct functional enrichment analysis of these differentially expressed (DE) genes [23]. The results showed that these DE genes were enriched in mitochondrial dysfunction (*p* = 5.5 × 10^−22^), oxidative phosphorylation (*p* = 4.41 × 10^−18^), and protein ubiquitination pathway (*p* = 5.23 × 10^−16^) (Table 2). Table 3 shows the top molecular and cellular functions of DE genes identified by IPA. The top five cellular functions that were affected by HGK suggest that the major activity of HGK is to affect the cell death and survival. Upstream regulator analysis in IPA was used to predict the upstream transcriptional regulators from the dataset. The overlap value was used to predict the potential transcriptional regulator through the gene expression database. EGFR (overlap *p*-value = 1.22 × 10^−11^) was a potential upstream regulator that is involved the gene expression changes in H1975 cells treated with HGK at 50 μM. Figure 3A shows the putative function of EGFR regulator in the network. These analyses reveal that HGK might modulate EGFR regulator and, thus, affect cell proliferation and death pathways in H1975 cells.

### 2.5. The Effects of HGK on EGFR Expression and Downstream Pathways

A549, PC9, and H1975 cells were treated with HGK at 0, 25, and 50 μM, and examined for the expression of EGFR and EGFR-related signaling pathways to address whether HGK might modulate EGFR and its downstream signaling. As shown in Figure 3B,C, the levels of total EGFR were decreased in a dose-dependent manner in HGK-treated A549, PC9, and H1975 cells. The levels of phospho-EGFR (pEGFR) were similarly reduced in the HGK-treated PC9 and H1975 cells. Accompanied with reduced EGFR levels in HGK-treated cells, several EGFR-downstream signaling pathways, including phospho-STAT3 (pSTAT3), phospho-AKT (pAKT), and phospho-ERK (pERK), were also inhibited in HGK-treated PC9 and H1975 cells. These results indicate that HGK modulates EGFR and its downstream pathways in NSCLC cells. 

We examined the effects of HGK on the mRNA expression of EGFR to address whether the reduced expression of EGFR in the HGK-treated cells might be mediated by transcirptional downregulation of EGFR. The levels of EGFR mRNA stayed unchanged in all of HGK-treated NSCLC cells, as shown in Figure 3D. These results suggest that HGK might modulate EGFR by affecting the stability of EGFR protein. We postulate that HGK might modulate EGFR signaling by accelerating EGFR degradation, as HGK is predicted to affect protein ubiquitination pathway (*p* = 5.23 × 10^−16^) (Table 2) based on transcriptome analysis. H1975 cells were treated cycloheximide in the presence or absence of HGK, and the levels of EGFR were determined by Western blot, to determine the effects of HGK on the protein stability of EGFR. As shown in Figure 3E, the level of total EGFR gradually reduced in the absence of HGK treatment. However, the level of EGFR was rapidly decreased in HGK-treated H1975 cells. We examined the effect of proteasome inhibitor (MG132) on the EGFR stability in H1975 to address whether the instability of EGFR protein by HGK treatment might be due to proteasome-mediated degradation [24,25]. The treatment of H1975 cells with MG132 at 10 μM or lower concentrations had no effect on cell proliferation (Supplementary Figure S1A). In the presence of MG132 at 10 μM, the level of EGFR was no longer reduced following treatment of HGK at 50 μM in H1975 cells (Figure 3F). Under this experimental condition, the combination of MG132 and HGK treatment for 12 h had no effect on cell cytotoxicity (Supplementary Figure S1B) or apoptosis induction, as evidenced by the lack of procaspase 9 cleavage (Figure 3F). Collectively, these results suggest that HGK can promote proteasome-mediated degradation of EGFR in NSCLC cells, which leads to the inhibition of cell proliferation and cell death.

### 2.6. Antitumor Activity of HGK in a Xenograft Mouse Model

**Figure 4:**
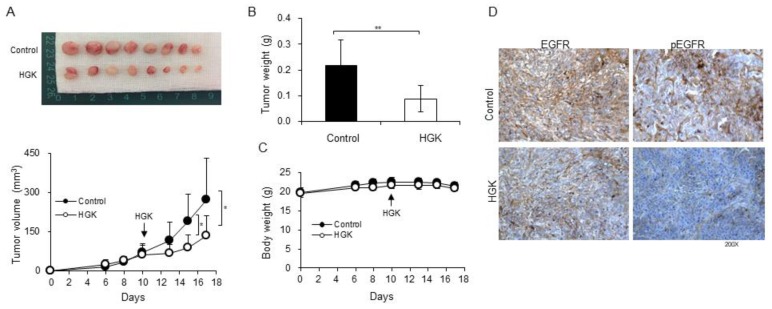
The antitumor effects of HGK in vivo. (**A**) The H1975 cells were inoculated subcutaneously in the right flank of nude mice. When the H9175 xenograft tumors reached around 50 mm^3^ at day 10 (as indicated by arrow), HGK was intraperitoneal injected every two days. The tumor volume was detected as described in Materials and Methods and is shown in the bottom panel. The excised xenograft tumors are shown in the upper panel. The effects of HGK treatment on the tumor net weights and body weights of mice are shown in (**B**) and (**C**), respectively. (**D**) Immunohistochemical staining of xenograft tumor sections for EGFR and phosphor-EGFR (pEGFR). The data that are shown in A B, C are presented as mean ± SD and they were analyzed by Student’s *t*-test. Asterisks denote statistically significant.

A xenograft mouse model was used in this work to evaluate the antitumor activity of HGK in vivo. The H1975 cells were subcutaneously inoculated in the right flank of nude mice. When the H9175 xenografts tumors reached around 50 mm^3^, we administered HGK (1.0 mg/kg body weight) in 100 μL of PBS by i.p. injection every two days. As shown in Figure 4A,B, the administration of HGK significantly inhibited tumor progression. There was no recognizable alteration in body weight or overt sign of toxicity in all of the treated mice (Figure 4C). IHC staining of the tumor xenografts showed the heavy staining of phospho-EGFR (pEGFR) and EGFR in untreated H1975 tumor xenografts (Figure 4D; upper panel), whereas weak staining of pEGFR and EGFR was detected in HGK-treated tumor (Figure 4D; lower panel). These results indicate that HGK displays antitumor activity against TKI-resistant NSCLC cells in vivo.

## 3. Discussion

In the study, we have isolated three flavonoid compounds from Genkwa Flos (Figure 1A), and have identified HGK as one of the active flavonoids that display anti-tumor activity against NSCLC cells. HGK displayed cytotoxic effects against NSCLC cells harboring constitutive activating EGFR mutations, including TKI-resistant H1975 and TKI-sensitive PC9 cells (Figure 1). Similar to the report by Wang et al., [20], HGK induced apoptosis in all of NSCLC cells that were used in this study (Figure 2B and Figure 3B,C). However, flow cytometric analysis of cell cycle progression revealed that HGK only induces cell cycle arrest in H1975 cells, but not in A549 and PC9 cells (Figure 2A). Therefore, the effects of HGK on cell cycle progression do not appear to be universal for all cancer cells. The discrepancy between the results of apoptosis shown in Figure 2A,B may reflect the different methods used for detecting apoptosis, i.e., an increase in the sub G1 phase (Figure 2A) versus phosphatidylserine exposure in the cell surface (Figure 2B), which detects an early event of apoptosis.

While the antitumor activity of HGK is likely related to its ability to induce apoptosis in cancer cells, the molecular basis for such an activity is not known. In this study, whole-transcriptome sequencing and Ingenuity Pathway Analysis (IPA) of the differentially expressed genes was used to analyze the molecular and cellular functions of DE genes and predict the upstream transcriptional regulators involved. These analyses suggest that HGK might affect certain factors that are involved in EGFR signaling and proteasome-mediated proteolysis (Figure 3A). Indeed, we have shown that HGK reduced the protein level of EGFR via proteasomal-dependent degradation (Figure 3B–F). As one of the most important post-translational modifications that facilitate EGFR degradation is ubiquitination [26] and that our analysis showed that the differentially expressed genes were enriched, including protein ubiquitination pathway (Table 2), it is likely that HGK might act to enhance the ubiquitination of EGFR and, thus, accelerates the proteasome-mediated process. The attachment of casitas B-lineage lymphoma (CBL), an E3 ubiquitin ligase, to activated EGFR is known to mediate the receptor degradation through the ubiquitin- proteasome system [27]. This raises the possibility that HGK may regulate CBL to enhance EGFR ubiquitination [24]. Future investigations are needed to test such a postulate.

Lastly, we have shown that HGK displays antitumor activity against xenografted H1975 cells in nude mice (Figure 4). HGK down-regulated EGFR and slowed down the growth of tumors, but not the total inhibition of tumor growth. Therefore, this compound might not be effective by itself in eradicating tumors, but it can be added as an adjuvant drug. For example, a combination of HGK and apigenin, a flavonoid compound that is commonly present in vegetables and fruits, has been shown to significantly increase the antitumor effects of apigenin in C6 glioma cells [20]. Therefore, HGK might hold capacity as an adjuvant drug in the treatment of NSCLC. As HGK is able to inhibit the tumor growth of TKI-resistant NSCLC cells (Figure 1 and Figure 4), the potential inclusion of HGK as an adjuvant drug in the treatment of NSCLC patients harboring activating EGFR mutations might be explored.

In conclusion, this study has identified HGK as one of the active flavonoids from Genkwa Flos that displays antitumor activity against NSCLC cells harboring activated EGFR mutations. Our molecular studies suggest a model that HGK functions by inducing the proteasome-mediated degradation of EGFR, thus inhibiting EGFR-downstream signaling and inducing apoptosis. The findings from this study may have potential applications in the future development of HGK as an anticancer agent that displays unique pharmacologic properties and anti-tumorigenesis activities against specific lung cancer patients.

## 4. Materials and Methods

### 4.1. Plant Material and Extraction

The Department of Pharmacy, Chang Gung Memorial Hospital at Chiayi, Taiwan, provided and authenticated dry flower buds of *Daphne genkwa*. A voucher specimen (No. CGU-DG-1) was deposited in the herbarium of Chang Gung University, Taoyuan, Taiwan [16]. The extraction of dried buds of *Daphne genkwa* Sieb. et Zucc. was as described in earlier work [16]. Briefly, dry flower buds of *Daphne genkwa* (5.0 Kg) were extracted with MeOH (30 L × 4) and then concentrated to give brown syrup (745.75 g). The syrup was suspended in H_2_O and partitioned first in CHCl_3_/water (1:1) and then in *n*-butanol/water (1:1), successively. The CHCl_3_ extract (511.19 g) was used in this study to isolate and identify the flavonoids that exhibit antitumor activity.

### 4.2. Isolation and Identification of Flavonoids

The CHCl_3_ extract was subjected to silica gel column chromatography by eluting with stepwise gradients of CHCl_3_: MeOH to obtain fourteen fractions. The fourth fraction was further chromatographed in the silica gel column by eluting with a mixture of n-hexane and acetone (3:1) to isolate 3′-methoxy genkwanin [18] (151.9 mg). The fifth fraction was further chromatographed in the silica gel column by eluting with a step gradients of CHCl_3_ and acetone to isolate genkwanin [10] (373.9 mg). The seventh fraction was further chromatographed in silica gel column by eluting with a step gradients of *n*-hexane and acetone to isolate HGK [17] (206.4 mg). The structures of these compounds were confirmed by comparison of their spectral data with the corresponding literature values. These compounds were dissolved in dimethyl sulfoxide (DMSO) to make a stock concentration at 100 mM and then stored at −20 °C before use.

### 4.3. Cell Lines and Culture

A549 and H1975 were obtained from the American Type Culture Collection (Manassas, VA, USA). Dr. Tzu-Chien V. Wang (Chang Gung University, Taoyuan, Taiwan) kindly provided the primary normal human foreskin fibroblasts (HFF3) and Dr. Pan-Chyr Yang kindly provided PC9 (National Taiwan University, Taiwan). A549 cells express wild-type EGFR; PC9 cells contain a deletion in exon 19 of *EGFR*; and, H1975 cells harbor two mutations (L858R and T790M) in *EGFR*. All of the cells were cultivated in RPMI-1640 medium containing 10% fetal bovine serum (FBS), 2 mM sodium pyruvate, 100 U/mL penicillin, and 100 U/mL streptomycin. The cells were grown at 37 °C in a humidified incubator containing 5% CO_2_.

### 4.4. Antibodies, Oligonucleotides, and Reagents

The culture media, FBS, and chemical compounds were purchased from Life Technologies (Grand Island, NY, USA). Antibodies against phospho-EGFR (Tyr1068), phospho-STAT3 (Tyr705), phospho-AKT (Ser473), phosphor-ERK (Thr202/Tyr204), caspase 9, and poly (ADP-ribose) polymerase (PARP) (Asp214) were purchased from Cell Signaling (Temecula, CA, USA). Anti-EGFR, anti-STAT3, anti-AKT, anti-ERK, and β-actin were purchased from Santa Cruz Biotechnology (Santa Cruz, CA, USA). Cycloheximide and MG132 were purchased from Sigma (St. Louis, MO, USA).

### 4.5. Assays for Viability, Cell Proliferation Capacity, Clonogenic Ability, and Apoptosis

Cell viability assay was carried out by plating 3000 cells/well into 96-well plates. In the following day, the cells were treated with various concentrations of HGK and then incubated for 24 h. Cell viability was measured while using MTT assay and staining with trypan blue. For MTT assay, 10 μL of MTT (5 mg/mL) solution was added to the cells in each well containing 100 μL of medium. After incubating at 37 °C for 3 h, the supernatant was removed and 200 μL of DMSO was added to the cells. The MTT color reaction was examined while using a microplate reader set at *A*560 nm. For trypan blue assay, the cells were trypsinized and stained with 0.4% trypan blue. The number of unstained viable cells was counted in a hemocytometer under microscope. The data were presented as means ± standard deviations from three independent experiments. The Student’s *t*-test was used for statistical analyses with the SPSS 16.0 software (IBM, New York, NY, USA). The unstained viable cells were determined by counting with a hemocytometer under microscope. The cell proliferation capacity was examined with an xCELLigence real-time cell analyzer (Roche Life Science, Indiana, USA) according to the manufacturer’s instructions [28]. For clonogenic ability, the cells were plated in six-well plates (500 cells/well). In the following day, the cells were treated with various concentrations of HGK for 24 h. The treated cells were then incubated in the absence of HGK for five days, stained with crystal violet, and the number of foci formation was determined. For apoptosis assay, the cells were seeded in six-well plates (1 × 10^5^ cells/well) with growth media. In the following day, the cells were placed in the media containing various concentrations of HGK for 24 h. Apoptosis was examined by the detection of phosphatidylserine exposure with Annexin V-FITC Apoptosis Detection Kit I (BD Biosciences, New Jersey, USA) [29], and by the cleaved PARP and caspase 9, as described previously [30].

### 4.6. Cell-Cycle Analysis

The cells were seeded in six-well plates (1 × 10^5^ cells/well) with growth media. In the following day, the cells were treated with various concentrations of HGK for 24 h. Cells was fixed in −20 °C absolute ethanol for 4 h and resuspended in 1 mL of PBS containing ribonuclease A at 20 μg/mL. After incubating at 37 °C for 30 min. and propidium iodide was added to each sample at 100 μg/mL. Cell cycle distribution was analyzed by flow cytometry (BD FACSCalibur TM system, Becton–Dickinson). 

### 4.7. RT-PCR, Western Blotting, and Immunohistochemistry (IHC)

The cells were seeded in six-well plates (1 × 10^5^ cells/well) with growth media. In the following day, the cells were treated with various concentrations of HGK for 24 h. The cell lysate were then collected for analysis by RT-PCR and Western blotting, as described previously [31]. IHC was performed, as described previously [29]. The primary antibodies used for staining were targeted against EGFR and pEGFR [32].

### 4.8. Whole-Transcriptome Sequencing

The total RNA from H1975 cells treated with and without HGK at 50 μM for 24 h was isolated while using Trizol® Reagent (Invitrogen, Massachusetts, USA), according to the manufacturer’s instruction. RNA was quantified at OD_260_nm by using a ND-1000 spectrophotometer (Nanodrop Technology, Wilmington, USA). The RNA 6000 LabChip® kit (Agilent Technologies, USA) was used with the Bioanalyzer 2100 (Agilent Technologies, Santa Clara, California, USA) to check the integrity and concentration of total RNA samples. RNA sample preparation for sequencing analysis was carried out according to the protocol that was provided by Illumina. Agilent’s SureSelect Strand Specific RNA Library Preparation Kit was used for library construction and 150–200 bp cDNAs were purified by AMPure XP Beads size selection (Beckman Coulter, High Wycombe, Bucks, UK). The DNA sequence was determined while using an Illumina Hiseq 2000 platform. 

### 4.9. Functional Enrichment Analysis 

The Ingenuity Pathway Analysis (IPA) (QIAGEN company, Redwood City, CA, USA), a web-based computational platform, was used to conduct functional enrichment analysis of genes [23]. A total of 2345 differential expression (DE) genes (>two fold change) were analyzed by the Core analysis enrichment tool that was based on gene expression database. Canonical pathways and Upstream Regulator Analysis found by core analysis in IPA are given with a *p*-value.

### 4.10. In Vivo Tumor Xenograft Study

The in vivo antitumor activity of HGK against human NSCLC was studied while using six-week-old nude BALB/c nu/nu male mice (*n* = eight per group). The animals were inoculated subcutaneously in the right flank with H1975 tumor cells (3 × 10^6^) in 100 μL on day 0. Drug treatment was started when the tumor volume reached around 50 mm^3^ on day 10. The mice were randomized into control and drug treatment groups with eight animals in each group. The powder of HGK dissolved in 100 μL of PBS was administered by intra-peritoneal (i.p.) injection (1.0 mg/kg body weight) every two days. The control group was treated with an equal volume of PBS. The tumor volume was monitored every two days while using calipers, and tumor volume was estimated according to the following formula: tumor volume = length × width^2^ /2. On day 17, the tumor-bearing mice was weighed and then sacrificed for the assay of tumor biology. The animals were also evaluated for body weights and consumption of food to access apparent signs of toxicity. All of the animal experiments were performed after obtaining the approval of the Institutional Animal Care and Use Committee (IACUC) of Chang Gung Memorial Hospital (IACUC approval no.: 2018031301, approval date: 6/19/2018).

### 4.11. Statistical Analysis

The presented results were representative of three independent experiments with similar results. Statistical differences was evaluated while using the Student’s *t*-test, and it was considered to be significant at *p* < 0.05.

## Figures and Tables

**Table 1:** Cytotoxic effects of the compounds Genkwanin, 3′-Methoxy genkwanin, and hydroxygenkwanin (HGK).

Name of Compounds (100 μM)	Cell Viability (%)	
	*HFF3*	*A549*
Genkwanin	86.46 ± 2.4	91.64 ± 11.0
3′-Methoxy genkwanin	55.15 ± 6.9	67.31 ± 5.0
Hydroxygenkwanin (HGK)	74.87 ± 3.4	22.03 ± 2.9

Cells were treated with the indicated compound at 100 μM for 24 h, and cell viability was analyzed by MTT assay. Data are expressed as mean ± SD of three independent experiments.

**Table 2:** Functional enrichment analysis of the differentially expressed genes in H1975 cells treated with HGK at 50 μM.

Ingenuity Canonical Pathways	*p*-Value
Mitochondrial Dysfunction	5.55 × 10^−22^
Sirtuin Signaling Pathway	1.29 × 10^−20^
Oxidative Phosphorylation	4.41 × 10^−18^
Protein Ubiquitination Pathway	5.23 × 10^−16^
Estrogen Receptor Signaling	4.34 × 10^−12^

**Table 3:** Top molecular and cellular functions of the differentially expressed genes in H1975 cells treated with HGK at 50 μM.

Molecular and Cellular Functions	*p*-Value
Cell Death and Survival	2.32 × 10^−6^ to 2.37 × 10^−42^
Cellular Development	2.05 × 10^−6^ to 2.40 × 10^−39^
Cellular Growth and Proliferation	2.05 × 10^−6^ to 2.40 × 10^−39^
RNA Post-Transcriptional Modification	8.49 × 10^−7^ to 2.98 × 10^−38^
Cell Cycle	2.26 × 10^−6^ to 1.61 × 10^−33^

## Supplementary Materials

The following are available online at https://www.mdpi.com/1420-3049/25/4/941/s1, Figure S1: The cell proliferation capacity of H1975 cells treated with MG132 or/ and HGK.
