# Supplementary figures and images for: Hydroxygenkwanin Suppresses Non-Small Cell Lung Cancer Progression by Enhancing EGFR Degradation
Source: Molecules. 2020 Feb 19;25(4):941. doi: 10.3390/molecules25040941 (PMC7070862; doi:10.3390/molecules25040941)

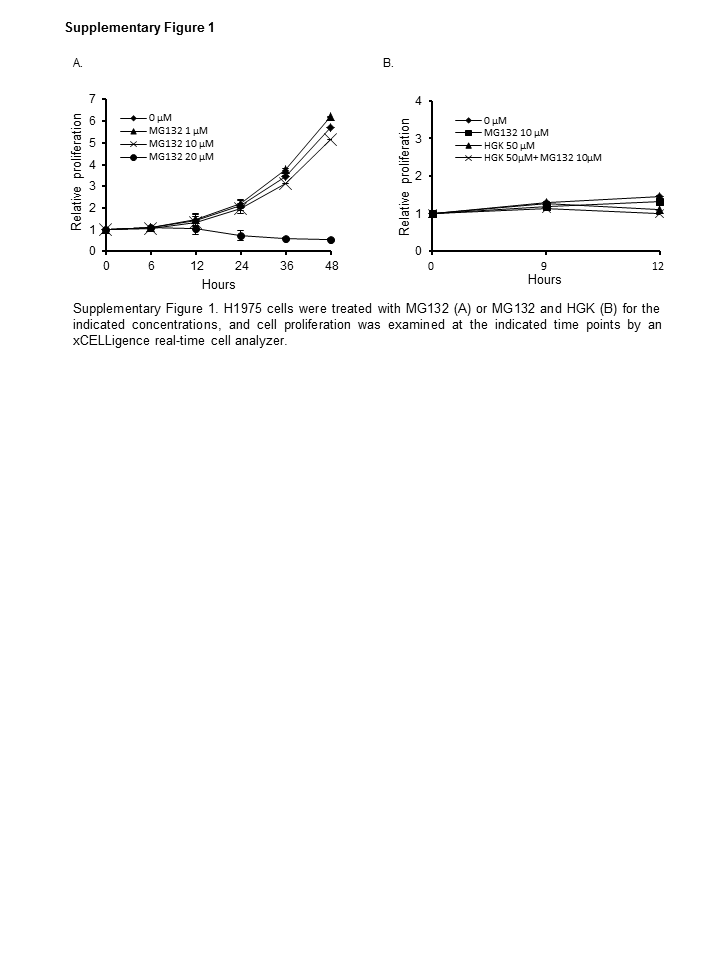

Supplement: Supplementary file 1 [file molecules-25-00941-s001.zip › molecules-715028-supplementary.tif]
